# Integrated transcriptome analysis of CSE1L regarding poor prognosis and immune infiltration in bladder urothelial carcinoma and experimental verification

**DOI:** 10.3389/fimmu.2024.1449251

**Published:** 2024-10-04

**Authors:** Runze Liu, Jiayi Ma, Yong Zhang, Zhongbao Zhou

**Affiliations:** ^1^ Department of Urology, Beijing Tiantan Hospital, Capital Medical University, Beijing, China; ^2^ Beijing National Day School, Beijing, China

**Keywords:** CSE1L, bladder urothelial carcinoma, prognosis, therapeutic target, immune infiltration

## Abstract

**Background:**

Bladder urothelial carcinoma (BLCA) is one of the most prevalent tumors globally, with its incidence rising notably in developed countries, significantly affecting human health. CSE1L encodes a protein that is involved in various cellular processes and plays a critical role in cancer initiation and progression. However, its role in BLCA remains underexplored.

**Methods:**

CSE1L expression in BLCA was analyzed using TCGA data and validated by qRT-PCR and Western blot in clinical samples. Survival analysis and Cox regression models were used to evaluate its prognostic value. Functional enrichment and protein interaction analyses were performed, and immune cell infiltration was assessed using CIBERSORT. Drug sensitivity was analyzed using GDSC data. *In vitro* assays evaluated the effects of CSE1L knockdown on cell proliferation, migration, and invasion.

**Results:**

CSE1L was found to be significantly overexpressed in BLCA tissues compared to normal tissues. High CSE1L expression was associated with poor overall survival and unfavorable clinicopathological features. Functional enrichment analysis revealed that DEGs related to CSE1L were involved in cell cycle regulation and immune-related pathways. Immune infiltration analysis indicated a significant correlation between CSE1L expression and various immune cell types, particularly T cells and macrophages. Drug sensitivity analysis identified several chemotherapeutic agents, including MG-132, Palbociclib, and Nutlin-3a, which were more effective in the low-CSE1L expression group, while the high-CSE1L expression group showed sensitivity to drugs like S-Trityl-L-cysteine, Bleomycin, and Cisplatin. *In vitro* knockdown of CSE1L in BLCA cell lines inhibited cell proliferation, migration, and invasion.

**Conclusions:**

The overexpression of CSE1L is associated with the progression and poor prognosis of bladder cancer, suggesting it could be a promising target for bladder cancer in the future.

## Introduction

Bladder urothelial carcinoma (BLCA) remains a significant global health concern, ranking 9th in incidence and 13th in cancer-related deaths worldwide (1). Treatment options for BLCA depend on various factors, including tumor stage, grade, and patient characteristics. Transurethral resection of bladder tumor (TURBT) is the standard treatment for non-muscle-invasive bladder cancer (NMIBC). For muscle-invasive bladder cancer (MIBC), radical cystectomy, with or without neoadjuvant chemotherapy, is often recommended (2, 3). Despite advances in understanding BLCA, challenges persist, including tumor heterogeneity, risk stratification, and treatment response prediction. Additionally, the identification of novel biomarkers, genetic alterations, and therapeutic targets holds promise for personalized medicine approaches.

CSE1L (chromosome segregation 1-like protein), also known as CAS (cellular apoptosis susceptibility protein), encodes a protein involved in various cellular processes and is found in humans and many other organisms. CSE1L is primarily recognized for its role in nucleocytoplasmic transport, the process by which molecules such as proteins and RNA move between the nucleus and the cytoplasm of a cell (4). CSE1L functions as part of the nuclear pore complex (NPC), serving as a gateway for the transport of molecules in and out of the nucleus (5). Additionally, CSE1L has been implicated in other cellular functions, including cell cycle regulation, cell division, and apoptosis (6, 7). Research on the CSE1L gene and protein has highlighted its importance in maintaining cellular homeostasis and its potential involvement in disease. Dysregulation of regulatory mechanisms can lead to aberrant CSE1L expression, contributing to developmental defects or disease progression (8). Further research is needed to deepen our understanding of the precise mechanisms by which CSE1L exerts its functions in development and disease. Unraveling the upstream regulators and downstream targets of CSE1L will provide insights into its molecular networks and potential therapeutic targets. Additionally, investigating the utility of CSE1L as a diagnostic or prognostic marker in BLCA may open avenues for personalized medicine approaches.

The present study aimed to explore the correlation between CSE1L and BLCA and identify potential biomarkers for this disease. The prognostic value of CSE1L expression was analyzed using survival analysis, and its correlation between CSE1L expression and clinicopathological characteristics in BLCA patients was examined.

## Materials and methods

### Data acquisition

Gene expression profile data and clinical information were collected from the TCGA-BLCA project, specifically utilizing the HTSeq-FPKM workflow. The data were sourced from the TCGA database (https://portal.gdc.cancer.gov/). To ensure a comprehensive analysis, RNA-seq data lacking clinical information were excluded, resulting in a total of 431 cases. These cases included both BLCA specimens and adjacent non-tumor specimens. Any unavailable or unknown clinical features were treated as missing values in the analysis.

### Survival analysis and construction of nomogram

Survival analyses of CSE1L were conducted using the R package *survival*. Patients were divided into low and high CSE1L expression groups. Univariate and multivariate Cox regression analyses of the clinical characteristics were used to establish a risk score as an independent prognostic predictor. To accurately estimate each patient’s prognosis, a nomogram was constructed based on the risk score and conventional clinical characteristics, including age, gender, stage, and TNM classification. The nomogram was then evaluated using calibration curves.

### GO and KEGG enrichment analyses

The *clusterProfiler* R package was used to identify significantly differentially expressed genes (DEGs) between BLCA samples and normal samples using an unpaired t-test. The Benjamini-Hochberg method was applied to adjust the threshold value, with an adjusted P <0.05 and |log FC| >1. Gene Ontology (GO) analysis revealed that these genes were associated with a diverse range of functional categories, including biological process and molecular function. Additionally, Kyoto Encyclopedia of Genes and Genomes (KEGG) enrichment analyses of DEGs were conducted using the Database for Annotation, Visualization, and Integrated Discovery (DAVID) online tools (https://david.ncifcrf.gov/), with a P-value cut-off criterion of <0.05.

### Composition of invasive immune cells in BLCA

The marker genes for 24 immune cell types were identified based on a literature review. The infiltration of these immune cells in BLCA was analyzed using the CIBERSORT algorithm and single-sample Gene Set Enrichment Analysis(ssGSEA) (9). The correlation between CSE1L expression and immune cell infiltration was assessed using the Wilcoxon method.

### Protein-protein interaction networks

The Pearson method was applied to calculate the correlation coefficient, with an adjusted |Cor| ≥ 0.3 and P ≤ 0.05. A protein-protein interaction (PPI) network was constructed using the STRING database (https://string-db.org) based on the intersection of genes between the screened DEGs and CSE1L-correlation genes. Subsequently, Gene Ontology Biological Process (GO-BP) enrichment analysis was performed on intersection genes, and the PPI network was annotated.

### Drug sensitivity analysis

Based on the Genomics of Drug Sensitivity in Cancer (GDSC, https://www.cancerrxgene.org/), we used the R package *prophytic* to predict the drug sensitivity of each tumor sample. The estimated half-maximal inhibitory concentration (IC50) of each compound was calculated. Representative drugs were then selected for Spearman correlation analysis.

### Patient samples

We collected BLCA tissues and matched normal bladder tissues from BLCA patients who underwent TURBT in the Urology Department of Beijing Tiantan Hospital, Capital Medical University, between February 2024 and May 2024. During the operation, the surgical specimens were promptly immersed in liquid nitrogen and stored at -80°C for subsequent use. All participants provided informed consent by signing the required form, and the project was approved by the Ethics Committee of Beijing Tiantan Hospital, Capital Medical University [KY2024-005-01].

### Cell lines and cell culture

The human normal urothelial cell line (SV-HUC-1) and BLCA cell lines (5637 and T24) were purchased from the Cell Bank of the Chinese Academy of Sciences. SV-HUC-1 cells were cultured in F-12K medium (Gibco), while T24 and 5637 cells were cultured in RPMI-1640 medium (Gibco). The medium was supplemented with 10% fetal bovine serum (FBS) and 1% penicillin and streptomycin. All cells were incubated at 37°C in a humidified environment with 5% carbon dioxide.

### Transfection

Small interfering CSE1L RNA (siR-CSE1L) and its corresponding negative control siRNAs (NC) were synthesized by General Biol Company (China). NC and siR-CSE1L were transfected into BLCA cell lines using Lipofectamine 3000 (Invitrogen, USA). All primer sequences are listed in **Table 1**.

**Table 1:** Primer sequences used in this research.

Name	Sequence (5’->3’)
GAPDH (human)-F	GTCTCCTCTGACTTCAACAGCG
GAPDH (human)-R	ACCACCCTGTTGCTGTAGCCAA
CSE1L(human)-F	GAACGGCTCTTTACTATGCGAGG
CSE1L(human)-R	CTGAAGAGCCAGGAAGTGTGAG
Si-CSE1L 1	GGATAATGTTATCAAAGTA
Si-CSE1L 2	CGACGGTATCAAATATATT

### RNA extraction, reverse transcription, and quantitative real-time PCR

Total RNA was extracted from freshly frozen tissues or bladder cancer cell lines using QIAGEN/74136/RNeazy Plus Mini Kit. Reverse transcription was then performed using the Vazyme/R312-02/HiScript III 1st Strand cDNA Synthesis Kit (+gDNA wiper). Subsequently, qRT-PCR was carried out using Vazyme/Q712-03/Taq Pro Universal SYBR qPCR Master Mix. The housekeeping gene GAPDH was used as an internal reference, and the relative expression level of the target gene was calculated using the 2−ΔΔCT method. Each experiment was performed in three replicates, and the primer sequences used are provided in **Table 1**.

### Western blot

Protein extraction and Western blotting were performed as follows. Tumor tissue was cut into small pieces, each weighing approximately 30mg, and placed in 500 μL RIPA lysis buffer (pre-added with protease and phosphatase inhibitors). A sample of 20 μg of protein was then loaded onto an SDS- PAGE gel and run under standard conditions, with the same volume of culture medium used as a control. The proteins were transferred to a PVDF membrane using iBlot 2 system following the standard protocol. The membrane was blocked with 5% nonfat milk in TBST and incubated for 1 hour at room temperature, then incubated overnight at 4°C with agitation in a solution containing the primary antibody diluted to the recommended concentration in TBST. After washing the blot in TBST three to four times for 5 minutes each at room temperature, the membrane was incubated with a horseradish peroxidase (HRP)-conjugated secondary antibody, diluted to the recommended concentration, for 1 hour at room temperature. Protein bands were visualized using a Clinx Gel Documentation and Analysis instrument, while ImageJ was used for the analysis of the gray values of the bands.

### Cell counting kit-8 assay

5637 and T24 cells transfected with NC and siR-CSE1L were plated in 96-well plates at a density of 5×10^3 cells per well. The cells were pre-cultured in a CO_2_ incubator at 37°C for 24 hours. CCK-8 reagent was added at 24, 48, 72 and 96 hours. After a 2-hour incubation at 37°C, absorbance at 450nm was measured using a spectrophotometer.

### Cell migration assay

Cells were seeded at a density of 2×10^5 cells per well in a 6-well plate and cultured for 24 hours until they adhered. A P200 pipette tip was used to create a vertical scratch. The cells were then washed three times with PBS to remove the dislodged cells, and images were captured immediately using a microscope (0h point). Afterward, serum-free medium was added, and the cells were incubated for an additional 12 hours before capturing images at the same locations. The results were analyzed using ImageJ software, with the wound healing area as an indicator of cell migration.

### Transwell invasion assay

For the cell invasion assay, the transwell plate with an 8-mm diameter polycarbonate membrane was used. The membrane in the upper chamber was coated with a layer of Matrigel diluted in medium, and the cells were resuspended in serum-free medium. The lower chamber was filled with complete medium. After incubation at 37°C in a humidified atmosphere with 5% carbon dioxide for 24-72 hours, the cells on the lower surface of the membrane were fixed with 100% methanol, stained with 0.1% crystal violet for 15-30 minutes, and counted under the microscope.

### Statistical analysis

The associations between clinical factors and CSE1L were analyzed using various statistical tests, including the Wilcoxon rank-sum test and logistic regression, and the t-test for numerical variables. The chi-square test was also employed for categorical data. Prognostic data were obtained from TCGA, and Cox regression analyses, along with the Kaplan-Meier method, were used to evaluate prognostic factors. In all tests, P value <0.05 was considered statistically significant.

## Results

### CSE1L expression in pan−cancer and BLCA

**Figure 1:**
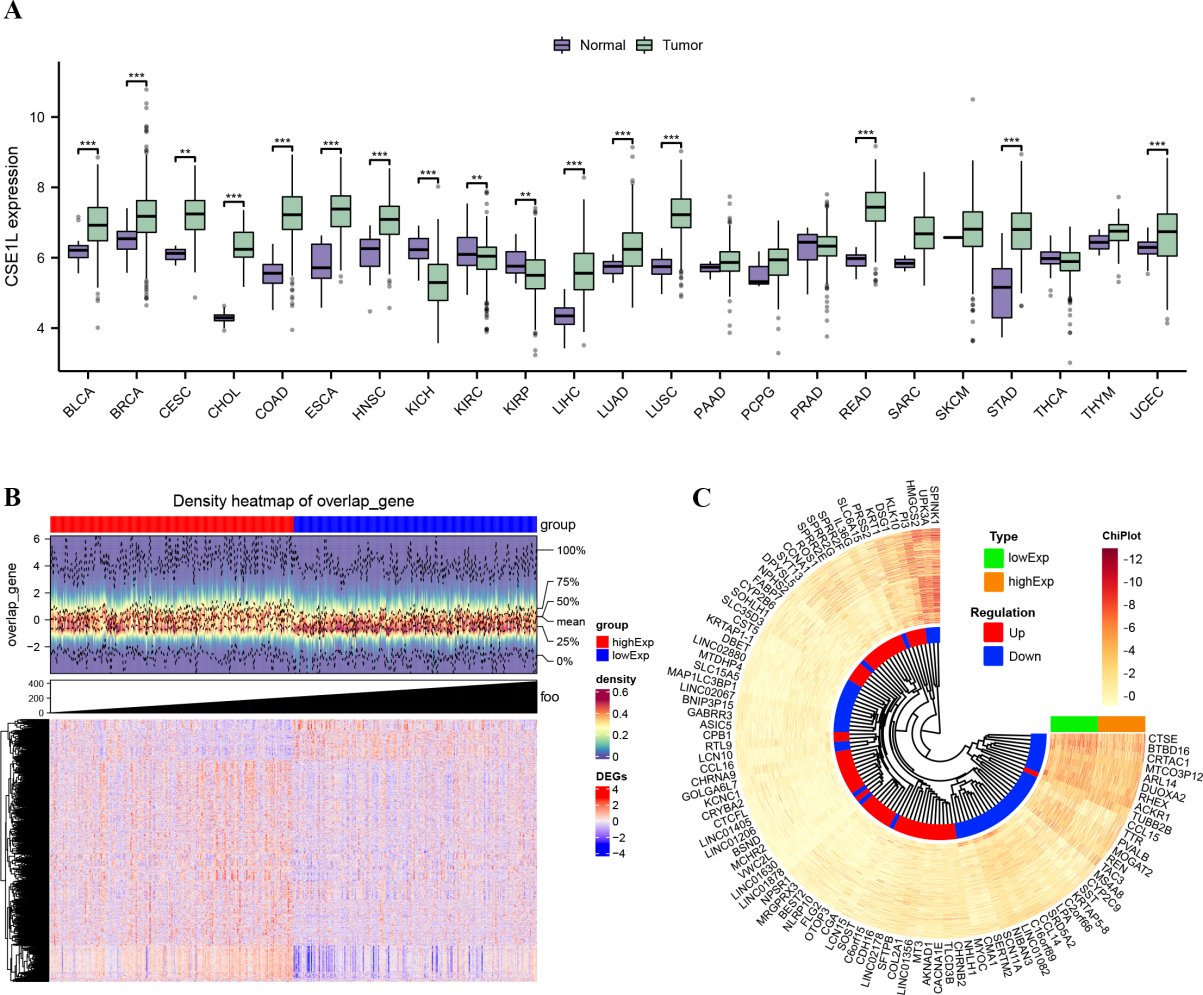
CSE1L expression in TCGA database and DEGs. **(A)** The mRNA expression levels of CSE1L in pan-cancer; **(B)** Density heatmap of DEGs; **(C)** Heatmap of top 100 DEGs. **p < 0.01; ***p < 0.001.

First, pan-cancer analyses were conducted to compare the CSE1L expression between tumor samples and their corresponding normal samples, using the Wilcoxon rank-sum test. Most human cancers, including BLCA, BRCA, CESC, CHOL, COAD, ESCA, HNSC, KICH, KIRC, KIRP, LIHC, LUAD, LUSC, READ, STAD and UCEC exhibit overexpression of CSE1L. In BLCA, a significant increase in CSE1L expression was observed in tumors compared to normal tissues (p < 0.05) (**Figure 1A**). Next, we compared the expression of CSE1L in 19 normal samples and 412 BLCA samples from the TCGA-BLCA dataset. CSE1L expression was significantly high in cancer samples, with a median level of 6.924 and 6.211 in tumor and normal tissues, respectively (p < 0.001) (**Figure 1B**). Subsequently, we grouped the samples based on high- and low-CSE1L expression groups, analyzed the DEGs, and generated heatmaps (**Figures 1B, C**).

### CSE1L could be an independent prognostic factor for BLCA

**Figure 2:**
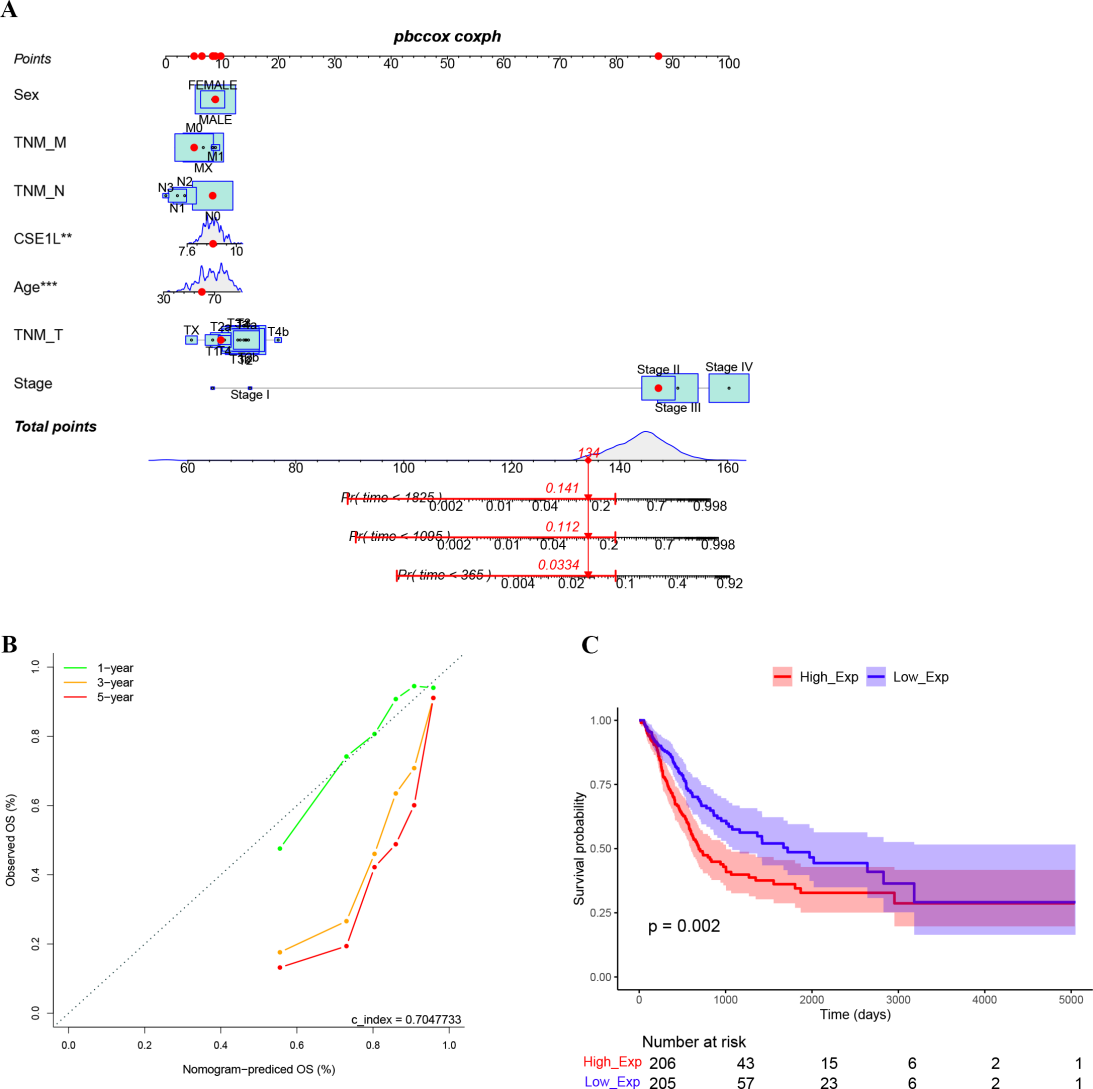
High expression of CSE1L indicates worse prognosis. **(A)** The nomogram to estimate the 1-, 3-, and 5-year OS of bladder cancer patients. **(B)** The calibration curves of actual values and predicted values provided by the nomogram. **(C)** Kaplan-Meier survival curves indicated that bladder cancer patients with high CASE1L mRNA expression had a shorter OS.

Clinical data and gene expression data for 412 BLCA samples were downloaded from TCGA database. We investigated the clinicopathological characteristics of BLCA patients with differential CSE1L expression. As shown in **Table 2**, compared with the low-CSE1L group, patients in the high-CSE1L group exhibited a higher proportion of histologic grade (P < 0.001), and worse primary therapy outcome (P = 0.003). There was also a significant correlation between CSE1L levels and smoking status (P = 0.013) as well as overall survival (OS) (P < 0.001). To explore the correlation between CSE1L expression and BLCA prognosis, we compared the survival rates of high-and low-CSE1L expression groups using Kaplan-Meier analysis. The results showed that high CSE1L expression was significantly associated with poor OS in BLCA patients (P=0.002) (**Figure 2C**).

**Table 2:** Clinical pathological characteristics of the patients.

Characteristics	Low expression of CSE1L	High expression of CSE1L	P value
n	206	206	
Pathologic T stage, n (%)			0.939
T1&T2	64 (16.9%)	59 (15.6%)	
T3	98 (25.9%)	98 (25.9%)	
T4	30 (7.9%)	29 (7.7%)	
Pathologic N stage, n (%)			0.682
N0	119 (32.3%)	119 (32.3%)	
N1	21 (5.7%)	25 (6.8%)	
N2&N3	45 (12.2%)	39 (10.6%)	
Pathologic M stage, n (%)			0.248
M0	109 (51.4%)	92 (43.4%)	
M1	4 (1.9%)	7 (3.3%)	
Pathologic stage, n (%)			0.824
Stage I&Stage II	68 (16.6%)	65 (15.9%)	
Stage III	68 (16.6%)	74 (18%)	
Stage IV	69 (16.8%)	66 (16.1%)	
Primary therapy outcome, n (%)			0.003
CR	133 (37.5%)	100 (28.2%)	
PR	13 (3.7%)	9 (2.5%)	
SD	9 (2.5%)	21 (5.9%)	
PD	27 (7.6%)	43 (12.1%)	
Gender, n (%)			0.823
Male	153 (37.1%)	151 (36.7%)	
Female	53 (12.9%)	55 (13.3%)	
Age, n (%)			0.691
<= 70	114 (27.7%)	118 (28.6%)	
> 70	92 (22.3%)	88 (21.4%)	
Histologic grade, n (%)			< 0.001
Low grade	19 (4.6%)	2 (0.5%)	
High grade	186 (45.5%)	202 (49.4%)	
Lymphovascular invasion, n (%)			0.082
No	57 (20.3%)	72 (25.6%)	
Yes	83 (29.5%)	69 (24.6%)	
Smoker, n (%)			0.013
No	66 (16.5%)	43 (10.8%)	
Yes	135 (33.8%)	155 (38.8%)	
OS event, n (%)			< 0.001
Alive	132 (32%)	98 (23.8%)	
Dead	74 (18%)	108 (26.2%)	

Bold values indicate P < 0.05, representing statistically significant differences.

A nomogram model was constructed using multivariate Cox regression based on the relative expression of the key gene CSE1L and its main clinical observation indicators. The results indicate that CSE1L is an independent prognostic factor in patients with BLCA, with the prognosis worsening as the risk score increased (**Figure 2A**). The calibration curve demonstrated that the actual OS values were consistent with those predicted by the nomogram, with an overall concordance index of 0.704 (**Figure 2B**).

### Functional enrichment and analyses

**Figure 3:**
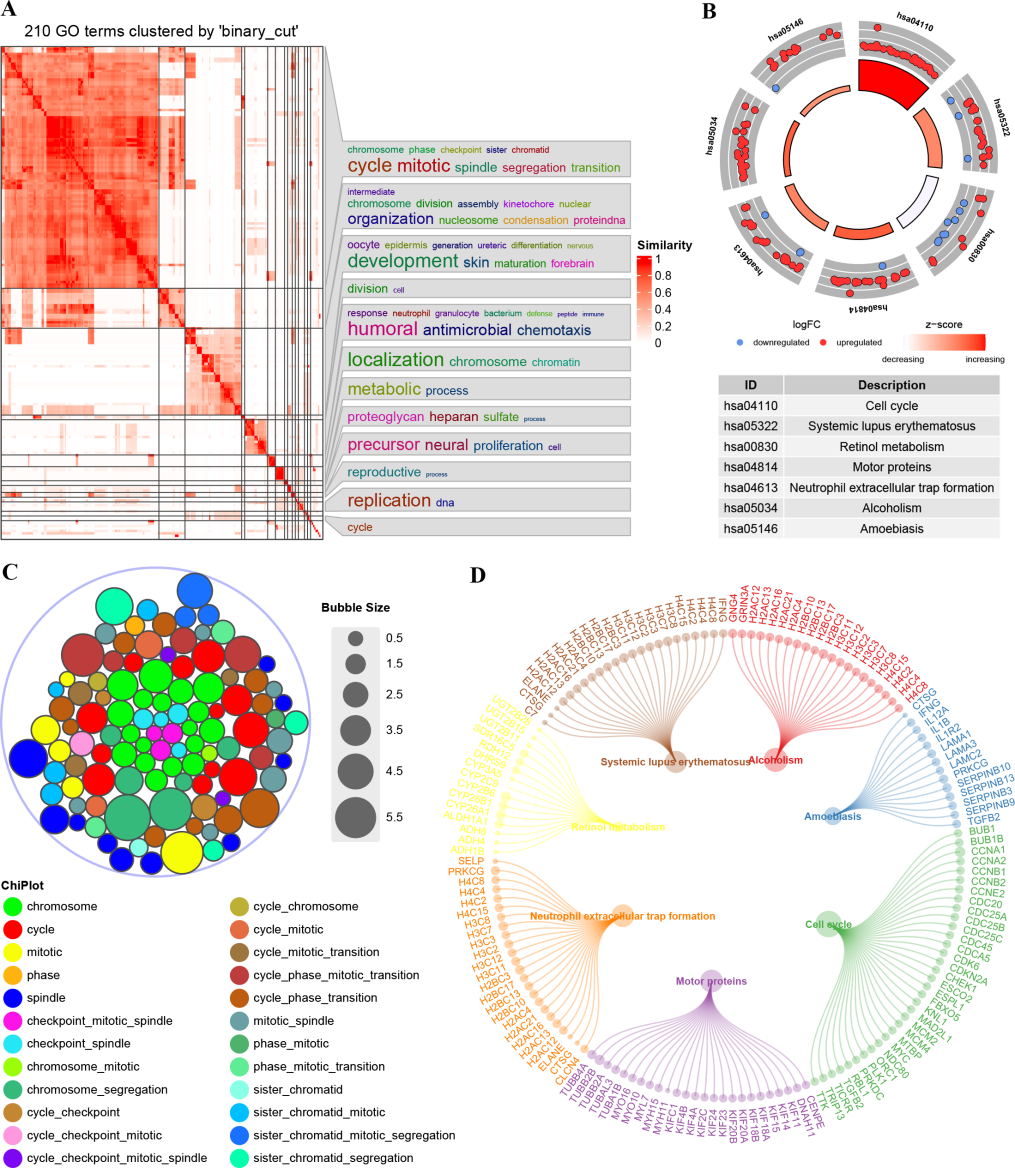
Enrichment plots from GO-KEGG analysis. **(A)** GO enrichment semantic similarity matrix. **(B)** Stacked chart of GO terms; **(C)** KEGG enrichment significance and the descriptions of each corresponding pathway. **(D)** DEGs enriched under the corresponding pathways.

We performed DEGs analysis using data from the TCGA cohort. The GO enrichment for DEGs showed that they were primarily associated with chromosome activity, the cell cycle, and mitosis (**Figures 3A, C**). The Kyoto Encyclopedia of Genes and Genomes (KEGG) enrichment analysis indicated significant enrichment in pathways related to cell cycle, systemic lupus erythematosus, retinol metabolism, motor proteins, neutrophil extracellular trap formation, alcoholism, and amoebiasis (**Figure 3B**). **Figure 3D** illustrates the DEGs enriched in corresponding pathways. Overall, the functions of DEGs are significantly related to the cell cycle.

### PPI network analysis

**Figure 4:**
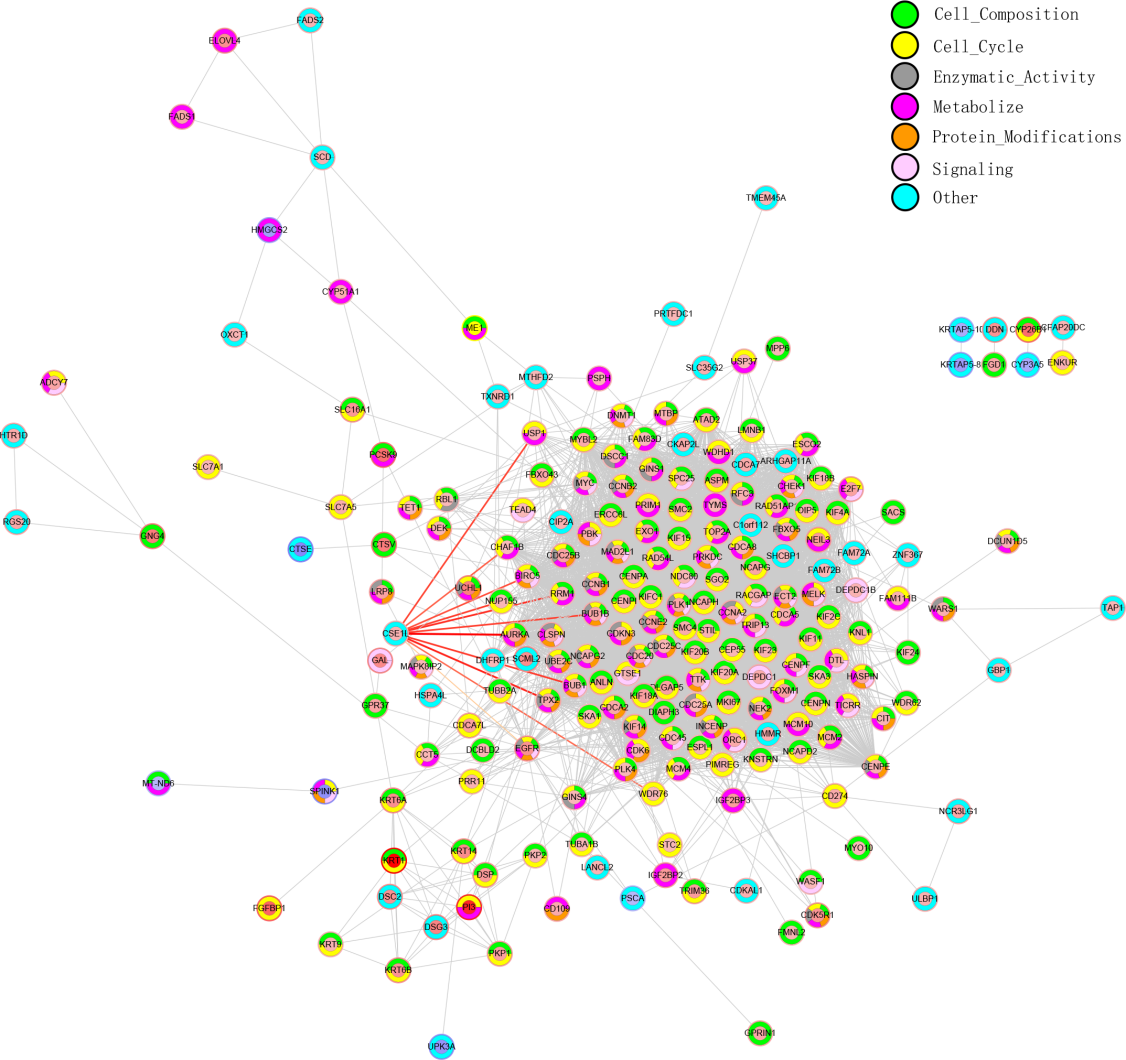
Protein-protein interaction (PPI) network of the intersecting genes.

PPI network analysis is essential for studying the pathway involved in tumor development. To better understand the potential biological functions of CSE1L in BLCA, gene correlations were assessed using Pearson correlation analysis. Using the STRING database, we constructed a PPI network. Based on the intersecting genes, we performed Gene Ontology Biological Process (GO-BP) enrichment analysis and classified their functions into seven categories for PPI network annotation. The inner circle in the PPI network represents the regulation of gene expression, while the outer circle indicates the different functions of the gene/protein (**Figure 4**).

### Correlation between CSE1L expression and immune infiltration

**Figure 5:**
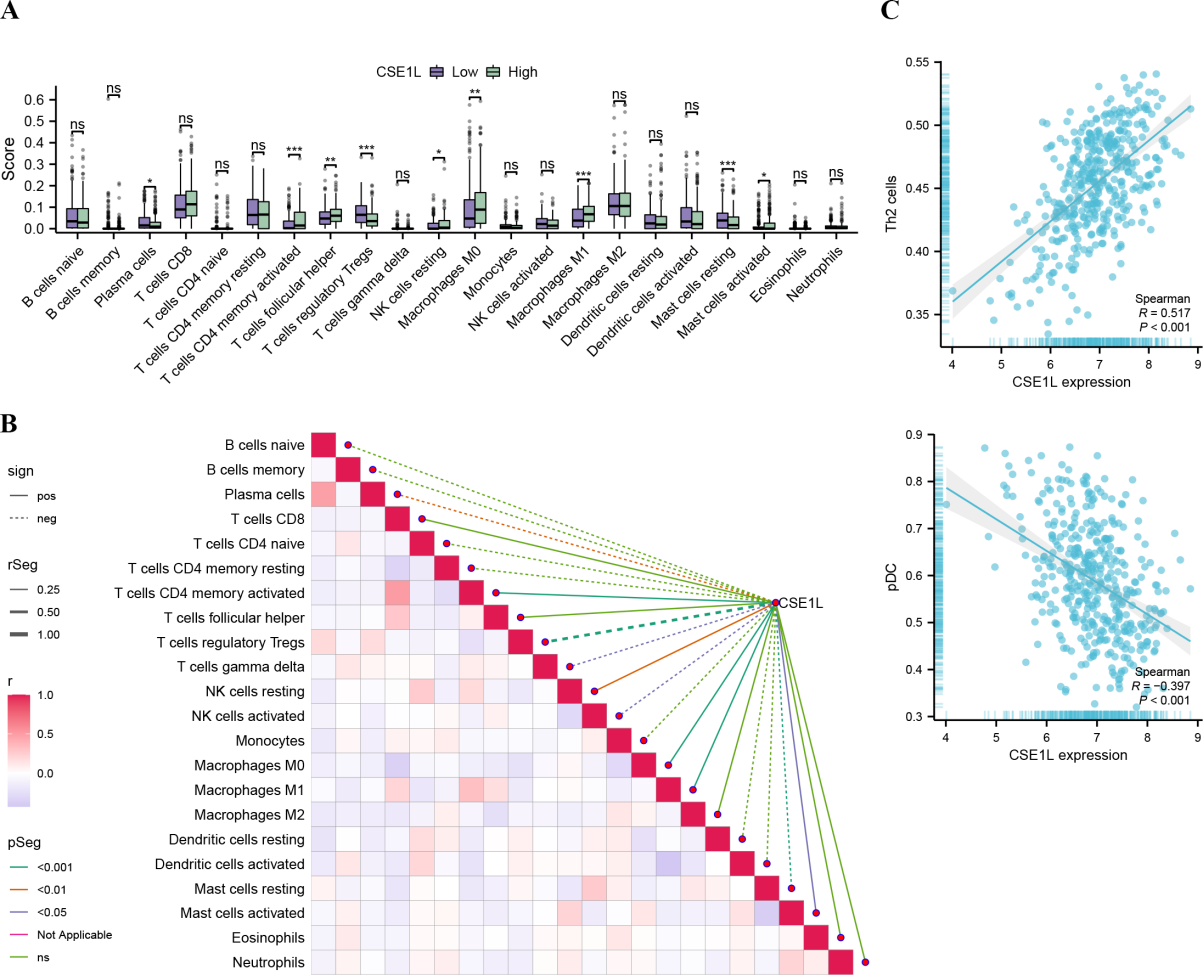
Association analysis of CSE1L gene expression and immune infiltration **(A)** Comparison of immune cells between high- and low-CSE1L expression groups (*P<0.05, **P<0.01, ***P<0.001.). **(B)** Immuno-infiltration correlation heat map and association with CSE1L gene expression **(C)** Correlation between CSE1L expression and Th2, pDC based on ssGSEA.

First, the CIBERSORT method was used to assess the infiltration of 22 different immune cell types in BLCA. The Wilcoxon method was employed to explore differences in immune cell infiltration between the high- and low-CSE1L expression groups. Our analysis revealed that CSE1L expression primarily affected T cells, macrophages, and mast cells (**Figure 5A**). We further analyzed the correlation between CSE1L expression and immune infiltration. As illustrated in **Figure 5B**, CSE1L expression was positively correlated with the CD4 memory-activated T cells, M0 macrophages, M1 macrophages, and resting NK cells, while it was negatively correlated with the infiltration level of regulatory Tregs and plasma cells. Next, we used ssGSEA to determine the infiltration of different immune cell types in BLCA. Spearman analysis was conducted to investigate the relationship between CSE1L expression and immune cell infiltration. Our analysis revealed that CSE1L expression was negatively correlated with the pDCs and CD56bright NK cells and positively correlated with the infiltration of Th2 cells (**Figure 5C**).

### Drug sensitivity analysis

**Figure 6:**
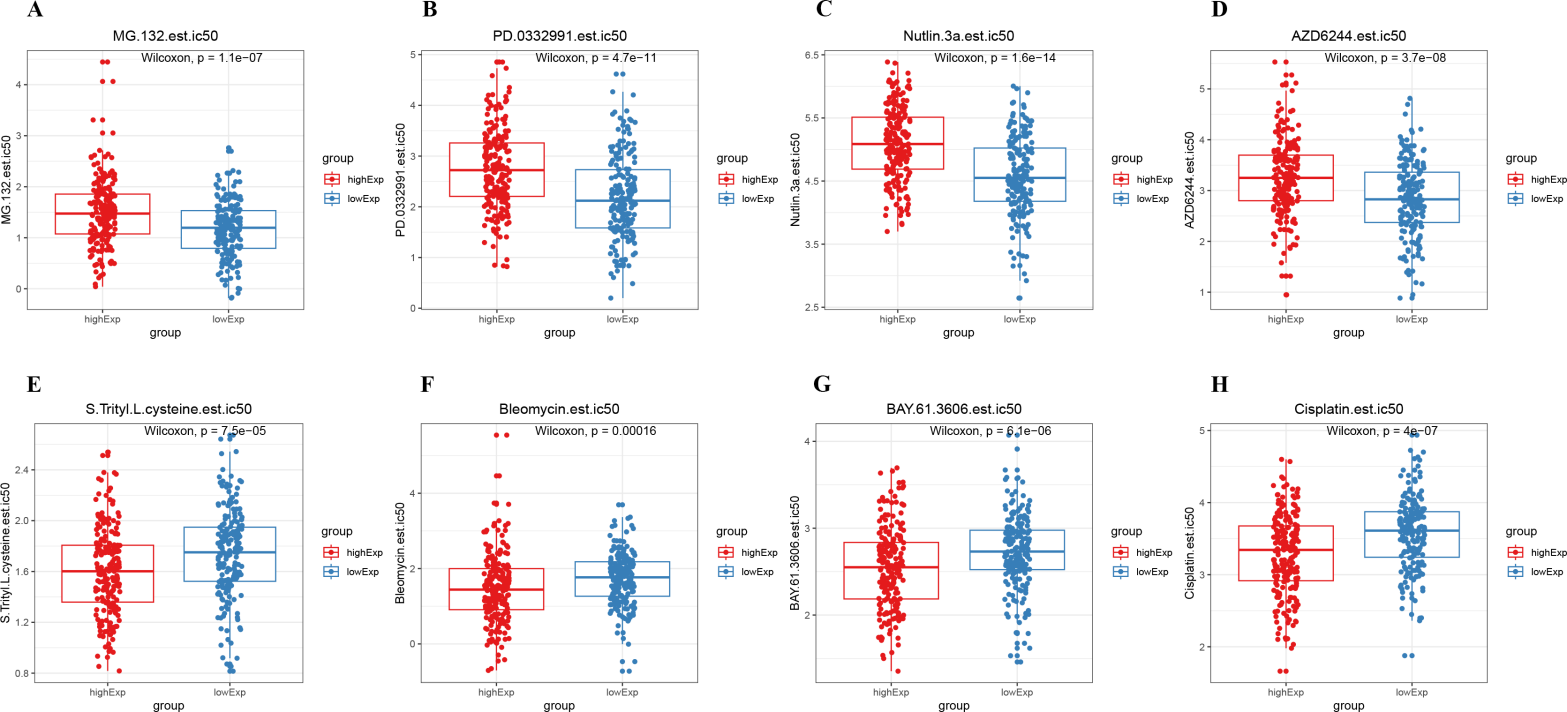
Drug sensitivity analysis. **(A–H)** There were significant differences in IC50 between the high and low CSE1L expression groups.

We conducted a chemosensitivity analysis of the TCGA dataset using the GDSC database through the R software package *prophytic*. The relationship between CSE1L expression levels and drug sensitivity was explored by calculating the correlation between the half-maximal inhibitory concentration (IC50) of chemotherapeutic drugs and CSE1L expression. A total of 70 drugs with IC50 values significantly correlated with CSE1L expression (p < 0.05) were identified. The results showed that the low CSE1L expression group was more sensitive to MG-132, Palbociclib (PD-0332991), Nutlin-3a, and Selumetinib (AZD6244), while the high CSE1L expression group was more sensitive to S-Trityl-L-cysteine (NSC 83265), Bleomycin, BAY 61-3606, and Cisplatin. These findings have implications for selecting specific medications based on anti-tumor drug sensitivity (Spearman │Cor│> 0.2 and p < 0.05, **Figures 6A–H**).

### CSE1L expression in BLCA tissues and cell lines

**Figure 7:**
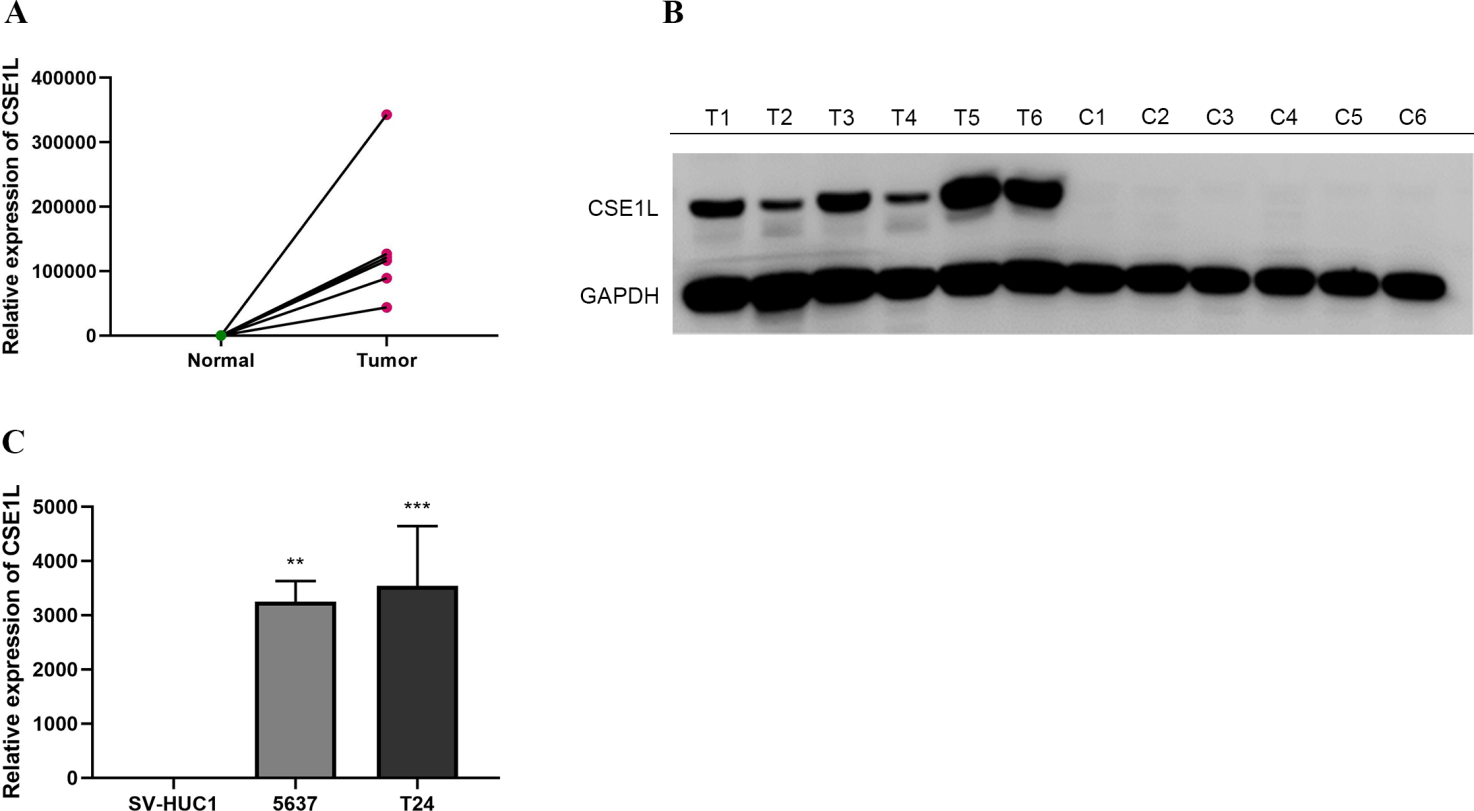
mRNA expression levels of CSE1L in BLCA tissues and cell lines. **(A, B)** mRNA expression levels of CSE1L in BLCA tissues; **(C)** mRNA expression levels of CSE1L in BLCA cell lines. (**p < 0.01, ***P < 0.001).

We collected tumor tissues and adjacent normal tissues from six pairs of BLCA patients for qRT-PCR and Western blot validation. The results revealed a significantly elevated expression level of CSE1L mRNA in the tumor tissues (**Figures 7A, B**). This finding was similarly confirmed in BLCA cell lines. Compared to the normal urothelial cell line SV-HUC1, CSE1L mRNA expression was significantly elevated in all BLCA cell lines tested (**Figure 7C**: 5637, P < 0.01; T24, P < 0.001).

### CSE1L exerted a promoting effect on BLCA *in vitro*

**Figure 8:**
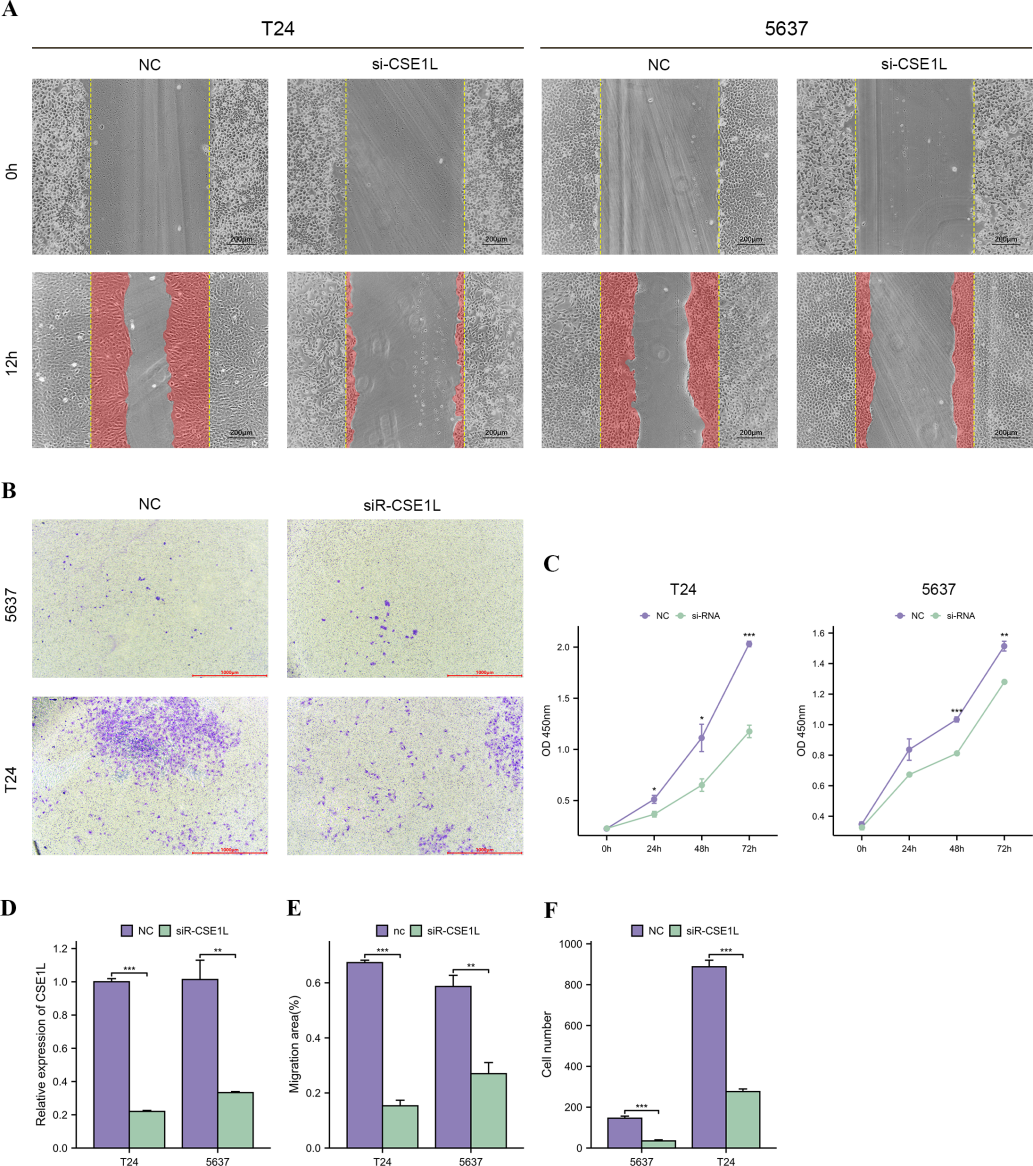
CSE1L promotes bladder cancer cells proliferation, migration and invasion *in vitro*. **(A, E)** Wound healing assay indicated that knockdown of CSE1L reduced the migratory ability of bladder cancer cells; **(B, F)** Transwell invasion assay indicated that knockdown of CSE1L reduced the invasive activity of bladder cancer cells; **(C)** CCK-8 assay indicated that knockdown of CSE1L reduced the proliferation ability of bladder cancer cells; **(D)** The mRNA expression level of CSE1L measured by qRT-PCR after cell transfection. *p < 0.05; **p < 0.01; ***p < 0.001.

To explore the biological functions of CSE1L in BLCA, we transfected 5637 and T24 cell lines with siR-CSE1L and a negative control (NC) separately. Knockdown efficiency was validated by qRT-PCR, which demonstrated a significant decrease in CSE1L expression at the mRNA level (**Figure 8D**). We then assessed the impact of CSE1L on the migration and invasion of BLCA cells using wound healing and transwell invasion assay. The wound healing assay indicated a significant reduction in the migration ability of 5637 and T24 cells after CSE1L knockdown (**Figures 8A, E**). Similarly, the transwell invasion assay showed a decrease in the invasive ability of BLCA cells as CSE1L expression was downregulated (**Figures 8B, F**). These results indicate that CSE1L promotes the migration and invasion of BLCA cells *in vitro*. Additionally, we investigated the effect of CSE1L on the proliferation of BLCA cells using CCK-8 assay. The CCK-8 assay showed that silencing CSE1L significantly attenuated the proliferation of 5637 and T24 cells (**Figure 8C**). In summary, these findings suggest that CSE1L promotes the development of BLCA *in vitro*.

## Discussion

Bladder cancer is the fifth most common cancer worldwide, characterized by a high incidence, poor prognosis, and a significant likelihood of recurrence. Currently, surgical resection is the most effective treatment (10). However, due to the high recurrence rate and substantial heterogeneity of bladder tumors, there is an urgent need for new molecular markers to predict patient survival in this disease.

In 1995, Brinkmann et al. first isolated a CSE1L-DNA fragment in breast cancer cells (11). CSE1L is the human homolog of the yeast gene CSE1, encoding a protein distributed in the cell nucleus and cytoplasm (12). The gene is located on 20q13, a site frequently amplified in various cancers and associated with genetic instability (13). CSE1L has been found to be involved in apoptosis, proliferation, survival, nuclear-cytoplasmic transport, and cancer metastasis (6, 14, 15). Additionally, CSE1L is a novel micro-vesicle membrane protein that may serve as a potential target for the development of efficient antibody-drug conjugates (ADCs) for cancer therapy (16).

As a key factor in the nuclear transport pathway, CSE1L participates in nucleocytoplasmic transport, a crucial process in tumor growth and development. Consequently, it may have applications in clinical diagnosis and treatment. Moreover, CSE1L expression is associated with tumor progression in various types of cancers. However, the clinical significance and biological functions of CSE1L in bladder cancer remain unclear. In this study, CSE1L was found to promote the initiation and progression of tumors. Evidence from the Kaplan-Meier plotter and nomogram analyses indicated that CSE1L possesses both diagnostic and prognostic value in distinguishing bladder cancer patients from healthy individuals. Furthermore, it was revealed that high CSE1L expression is associated with poor prognosis.

Based on the differentially expressed genes we screened and CSE1L-related genes, a PPI network was constructed. GO-BP enrichment analysis was performed on the intersecting genes, categorizing their functions into seven group: cell composition, cell cycle, enzymatic activity, metabolize, protein modifications, signaling and others. Genes that primarily interact with CSE1L include AURKA, NUP155, BIRC5, and TPX2. Interestingly, many of these genes are associated with unfavorable patient prognosis. Our study also revealed that CSE1L expression in bladder cancer is correlated with the type and density of infiltrating immune cells. The degree of infiltration of various immune cells, including T cells and macrophages, was significantly correlated with the CSE1L expression. CSE1L is believed to play a role in nucleus-to-cytoplasmic transport, chromosome separation during mitosis, proliferation, and apoptosis. In our study, the decrease in immune infiltration of regulatory T cells may be due to the high expression of CSE1L in these cells, leading to their apoptosis. The relationship between CSE1L expression and immune regulatory cells warrants further investigation.

Previous studies have shown that the expression of CSE1L is positively correlated with the immune checkpoint molecules PD-L1 (CD274) and PDL2 (PDCD1LG2). Progression-free survival (PFS) was significantly shorter in patients with high CSE1L expression compared to those with low expression, suggesting that CSE1L expression may affect the therapeutic efficacy of PD-1 monoclonal antibodies (17). Studies have also indicated that PD-L1 protein levels are reduced following CSE1L silencing (18). Additionally, Lee et al. found that serum phospho-CSE1L could be used for early detection of tumor-targeted therapy efficacy and to monitor secondary resistance in mouse tumor xenograft models. The CSE1L high expression group was more sensitive to S-Trityl-L-cysteine (NSC 83265), Bleomycin, BAY 61-3606, and Cisplatin, while the CSE1L low expression group was more sensitive to MG-132, Palbociclib (PD-0332991), Nutlin-3a, and Selumetinib (AZD6244). Notably, Nutlin-3a, which inhibits the MDM2-p53 interaction and stabilizes p53 protein, induces autophagy and apoptosis. In future studies, we will verify these findings using a larger cancer cell panel and *in vivo* animal models.

In this study, a series of experiments was conducted to elucidate the biological functions of CSE1L in bladder tumor cells. We found that silencing CSE1L expression in bladder cancer cells inhibited cell proliferation and reduced cell invasion and migration. CSE1L promotes tumor progression by enhancing cell proliferation and contributing to drug resistance. These findings align with our understanding of CSE1L’s impact on cancer cell growth and are consistent with previous studies.

Multiple potential underlying mechanisms of CSE1L action have been documented in various types of cancer. Studies have proposed that CSE1L interacts with the cAMP/PKA and RAS/ERK signaling pathways in melanoma. According to Jiang et al., the phosphorylation of CSE1L is regulated by extracellular signal-regulated kinase (ERK) (19, 20). Notably, CSE1L promotes melanin formation and melanoma progression by influencing the expression and phosphorylation of CREB (cAMP response element binding protein) and MITF (microphthalmia-associated transcription factor) (20). The PKA signaling pathway induces CREB phosphorylation, thereby prompting MITF expression. Furthermore, the PKA pathway also interacts with the MAPK/ERK pathway, leading to ERK activation. ERK, in turn, interacts with CSE1L, facilitating the phosphorylation of MITF (21). In gastric cancer, CSE1L inhibition has been shown to activate the PI3K/AKT/mTOR and MEK/ERK pathways by downregulation of MITF and GPNMB (15). In pancreatic cancer, CSE1L may regulate proliferation through the AKT signaling pathway (22). In lung cancer, CSE1L interacts with p65 and regulates MAPK signaling (23, 24). CSE1L could also promotes the proliferative and migratory abilities in oral cancer by positively regulating MITF and activating the Akt/mTOR pathway (25). In osteosarcoma cells, CSE1L, a positive regulator of MSH6 protein, is associated with poor patient prognosis. A rescue study suggested that the inhibitory effect of CSE1L knockdown on osteosarcoma cell growth could be reversed by overexpression of MSH6 (26). Current research has investigated whether CSE1L can be used to predict the prognosis of bladder cancer (BLCA) and provide treatment recommendations. However, this study had several limitations. First, due to the lack of additional data sources, the study may be subject to bias. Second, prospective studies are needed to validate our findings. Third, the stability and accuracy of CSE1L as a prognostic marker must be verified using data from other sources. Fourth, the molecular mechanisms by which CSE1L influences the development and progression of BLCA require further exploration through basic experiments.

## Conclusions

The expression level of CSE1L is upregulated in BLCA cells and is correlated with tumor progression, poor prognosis, and immune infiltration in bladder cancer. *In vitro* experiments demonstrated that the proliferation, migration, and invasion of BLCA cells can be inhibited by knocking down CSE1L expression.

## Data Availability

The original contributions presented in the study are included in the article/supplementary materials. Further inquiries can be directed to the corresponding authors.
